# The Effect of the COVID Pandemic on Clinical Psychology Research: A Bibliometric Analysis

**DOI:** 10.3390/bs14060463

**Published:** 2024-05-30

**Authors:** Jordan Anders, Michael S. Vitevitch

**Affiliations:** Spoken Language Laboratory, Department of Speech Language Hearing: Sciences & Disorders, Dole Human Development Center, University of Kansas, Lawrence, KS 66045, USA

**Keywords:** COVID, men, women, mental health, network analysis, bibliometric analysis

## Abstract

The present bibliometric analysis used traditional measures and network science techniques to examine how the COVID-19 pandemic influenced research in Clinical Psychology. Publication records from the Web of Science (WoS) were obtained for journal articles published prior to (2015 and 2018), during (2020), and at the end of the pandemic (2022) for the search terms “men and mental health” and “women and mental health”. Network analyses of author-provided keywords showed that COVID-19 co-occurred with fear, anxiety, depression, and stress for both men and women in 2020. In 2022, COVID-19 co-occurred with topics related to world-wide lockdowns (e.g., alcohol use, substance use, intimate partner violence, loneliness, physical activity), and to more fundamental topics in Clinical Psychology (e.g., eating disorders and post-traumatic stress disorder). Although the COVID pandemic was associated with several changes in the research topics that were examined in Clinical Psychology, pre-existing disparities in the amount of mental health research on men compared to women did not appear to increase (in contrast to increases associated with COVID in pre-existing gender disparities observed in other areas of society).

## 1. Introduction

COVID-19 (coronavirus disease 2019) refers to a respiratory disease similar to the flu or pneumonia caused by the SARS-CoV-2 virus. The first human case of the disease was reported in November 2019, and spread rapidly around the world, leading to the World Health Organization (WHO) to declare a Public Health Emergency of International Concern in January 2020, and a pandemic in March 2020 [1]. The WHO declared an end to the Public Health Emergency in May 2023 [2]. In addition to the hundreds of millions of cases of and millions of deaths from COVID-19 world-wide [3], the disease and measures to contain its spread had widespread effects on social, political, economic (e.g., labor markets and supply chains), and educational institutions in countries around the world [4,5,6,7,8,9,10,11,12]. The disease and measures to contain its spread also affected various psychological and physical behaviors (e.g., eating, sleeping, and exercise) of individuals around the world [13,14]. Several studies of the “science of science” (described in more detail below) examined how the disease and measures to contain its spread influenced the behaviors of individual scientists in a range of disciplines [15,16]. The present science of science study examined how research in a specific scientific discipline—Clinical Psychology (a discipline that is relevant to the topic of this special issue: The Impacts of COVID-19 on Mental Health and Well-Being)—was influenced by COVID-19. 

The science of science uses various analysis techniques and measures to examine how science itself works [17], with the goal of discovering general properties, trends, or characteristics that might help science administrators and policy makers, scientists in an entire discipline, or individual researchers to advance scientific discovery [18]. Conventional statistical analyses (e.g., counts, means, percent change, etc.) provide science of science investigators with some insight. However, the mathematical, analytic, and visualization tools of network science reveal patterns and relationships that cannot be observed in standard tables or figures of data. In the network science approach, nodes represent individual entities (e.g., authors or journal articles), and connections are placed between nodes that are related in some way (e.g., co-authors on a publication, or an article that cited another article). The nodes and connections produce a web-like structure that can be examined at the micro-level (i.e., focusing on characteristics of individual nodes), the meso-level (i.e., focusing on characteristics of a sub-set of nodes), or the macro-level (i.e., focusing on characteristics of the entire network). 

An example of the network science approach being used to examine how science in general works is seen in [19], whose authors created a network containing over 1 million nodes (representing journal articles published in 7121 journals) with connections between nodes indicating that an article in one journal cited an article in another journal. Examination of the citation network at the macro-level revealed citation trends between various scientific disciplines, with biochemistry emerging as the most interdisciplinary field, acting as a “hub” that connected a number of other disciplines.

Focusing on just a single discipline, the researcher in [20] examined how the field of Speech-Language-Hearing Sciences and Disorders changed in the first 20 years of the 21st century. In this analysis, nodes in the network represented important terms that appeared in the articles published in the *Journal of Speech-Language-Hearing Research* (a leading journal in that discipline) during that time frame. Connections were placed between terms that co-occurred in a given article. Comparison of the networks of terms from articles published in 2001, 2005, 2010, 2015, and 2021 revealed that over time, the discipline increasingly drew on the theories and methods of other disciplines (e.g., Cognitive Psychology, Linguistics), extended the languages being studied beyond English, and expanded the number and type of disorders/conditions being treated by the discipline in that time frame. 

In the present bibliometric analysis, we used network analysis to examine possible changes in the research topics of a specific discipline (Clinical Psychology) that occurred over time. Rather than look at how a discipline slowly and organically evolved over a broad swath of time, as in [20], we instead examined how an acute and unanticipated event that disrupted a wide range of societal institutions might cause a sudden shift in the research topics that a given discipline focuses on. That is, we compared the topics being studied in Clinical Psychology shortly before the start of the COVID pandemic and shortly after the start of the COVID pandemic (cf., [21]). 

Previous science of science studies of science in general found that the COVID pandemic negatively affected levels of scientific and academic productivity in the science, technology, engineering, and mathematics (STEM) and medicine fields [16,22]. Compared to male scientists, the negative impacts of COVID disproportionately affected female scientists, who were faced with increased caregiving responsibilities to children and other family members. These findings suggest that the COVID crisis exacerbated existing gender inequalities in STEM and medical researchers, and might have long-term effects on their career development [15]. 

Previous studies looking at mental health issues more broadly found that the COVID pandemic lead to a 25% increase in the global prevalence of anxiety and depression [23]. Given this dramatic increase in mental health issues resulting from the COVID pandemic, we wanted in the present science of science analysis to focus on how the COVID pandemic might have shifted research topics in the field of Clinical Psychology rather than how COVID influenced research or research productivity in STEM and medicine more broadly. Further, given the gender disparities in research productivity observed in STEM and medical researchers that were exacerbated by the COVID pandemic [15,16], and gender disparities in mental health in general [24], we also considered in the present science of science analysis if the potential shift in research topics in Clinical Psychology that might be attributed to COVID might also differ for men and women. To examine these potential changes, we used conventional measures as well as techniques from network science (which provide mathematical tools to quantify potential changes) to perform a bibliometric analysis at the macro- and micro-levels to examine (among other things) how key terms found in published research articles on mental health issues in men and women prior to COVID might have changed after COVID.

## 2. Materials and Methods

Bibliometric records of peer-reviewed articles from journals categorized as “Clinical Psychology” with the keyword search terms “men and mental health” and “women and mental health” for the years 2015, 2018, 2020, and 2022 were downloaded (on 23-JUNE-2023) from the Web of Science (WoS) database, which is a commonly used database for bibliometric analyses that includes journals and conference proceedings from numerous disciplines in the physical sciences, social sciences, the arts, and humanities. Given the global impact that COVID had on mental health, we selected the year 2020 (the year the pandemic was declared). Given that the World Health Organization (WHO) and the U.S. Department of Health and Human Services (HHS) declared an end to the Public Health Emergency in May 2023 [2], we selected the year 2022, which was the year closest to the end of the pandemic in which a full year of publications could be obtained. Given that 2022 was two years after 2020 (the year of the pandemic), we also selected two years prior to the pandemic (i.e., 2018) and 5 years prior to the pandemic (i.e., 2015) in order to detect any trends that may have existed prior to, but been exacerbated by, the pandemic. 

For each year, a tab delimited text file was exported that contained a full record and cited references. JASP [25] and VOSViewer (version 1.6.20; [26]) were used to analyze and visualize the data in the following ways:

I. *Number (and percentage) of research articles related to men and to women for each year*. This value will allow us to observe any gender disparities in the amount of research conducted on men and women, and how such disparities might have changed across the years we examined. Conventional statistics can be used to assess changes in values over time (e.g., ANOVA, regression, etc.) or to assess changes in distributions (e.g., Chi-square).
II. *Networks of co-occurring author-provided keywords*. The author-provided keywords (henceforth “keywords”) were extracted from the Web of Science (WoS) records. Networks were created for the keywords that co-occurred each year for men and women. Nodes in the networks represented the keywords, and connections were placed between terms that co-occurred. To reduce the number of idiosyncratic terms and increase the interpretability of the visualizations, only terms that (co-)occurred more than *x* times per year were included in each network. The value of *x* was set to 3 for the men and mental health keyword networks and to 6 for the women and mental health keyword networks to account for the nearly 2 times the number of publications on women and mental health compared to men and mental health. This resulted in the top ~1% most frequently occurring keywords being examined for both men and women. The visualization and network analysis of keywords enables us to determine at the macro-level if there are patterns or trends in research topics related to mental health issues in men and women that may be present/absent. By comparing the networks across years, one can assess if these patterns or trends vary in prevalence for men and women over time.
III. *COVID-19 ego networks*. Given our interest in how COVID-19 influenced research on mental health in men and women, we also created for 2020 and 2022 what is known as an *ego network* for the keyword “COVID-19”. An ego network (short for egocentric network) is a type of network that includes a single node (an “ego”) and the nodes that are directly connected to it (called “alters”), allowing us to “zoom in” on a specified node. In the present case, the ego network would show which keywords were connected to “COVID-19” for the research on men and women published in 2020 and 2022. Visualizing the ego networks for the COVID-19 keyword enables us to focus on the micro-level to consider how this global health emergency directly influenced specific research topics related to mental health issues in men and women.
IV. *Measures of Centrality*. Measures of centrality are commonly used in network science to identify “important” nodes in a network, or nodes that facilitate or inhibit the flow of information/gossip/goods/disease in a network [27]. There are several measures of centrality [28], but two that are most useful for the keyword co-occurrence networks in the present study are degree centrality and closeness centrality. *Degree centrality* is the simplest measure of centrality, and refers to the number of connections incident to a node (for a more formal definition, see [27]). In the present case, degree centrality measures how many keywords co-occur with a given keyword. Nodes with many connections are considered more “important” than nodes with few (or no) connections. *Closeness centrality* refers to the number of connections it takes (on average) to go via the shortest path from a given node to all other nodes in the network (for a more formal definition, see [29]). If few connections (on average) must be traversed to get from node *x* to all the other nodes in a network, then that node is said to be *close* to all the other nodes in the network and would be considered more “important” than a node that is (on average) *far* from all the other nodes in the network (i.e., requiring the traversal of many connections on average). Measures of centrality tend to be correlated (though not perfectly).

To facilitate replication and encourage further exploration of the data, the WoS files and .net files (a format commonly used for network analysis and visualization software) used to create the keyword networks and ego networks are available at an OSF website (https://osf.io/95yhd/). A tutorial on how to use VOSViewer can be found in [20]. 

## 3. Results

Table 1 shows the number (and percent) of articles obtained for “men and mental health” and “women and mental health” from WoS for the years examined. A Chi-square analysis (χ^2^ = 3.35, *p* = 0.07) shows that the changes in the number/percentage of published articles related to mental health for men and women over the years examined were not statistically significant at conventional *p*-values (i.e., *p* < 0.05). Further, the increase of ~1.7 times the number of articles published over the years examined (i.e., 2015 to 2022) is within the expected increase in scientific publication (i.e., a doubling of output every 15 years) observed for science in general [17]. Thus, the apparent changes in the amount of research being conducted on mental health issues for men and women in the timeframe we examined are consistent with the changes in the amount of research being conducted that one might expect for science in general. 

Networks containing keywords as nodes and connections placed between terms that co-occurred together *x* times (*x* = 3 for men, *x* = 6 for women) were constructed for men and women for each year examined. Nodes represented the keywords, and connections were placed between keywords that co-occurred. 

**Figure 1:**
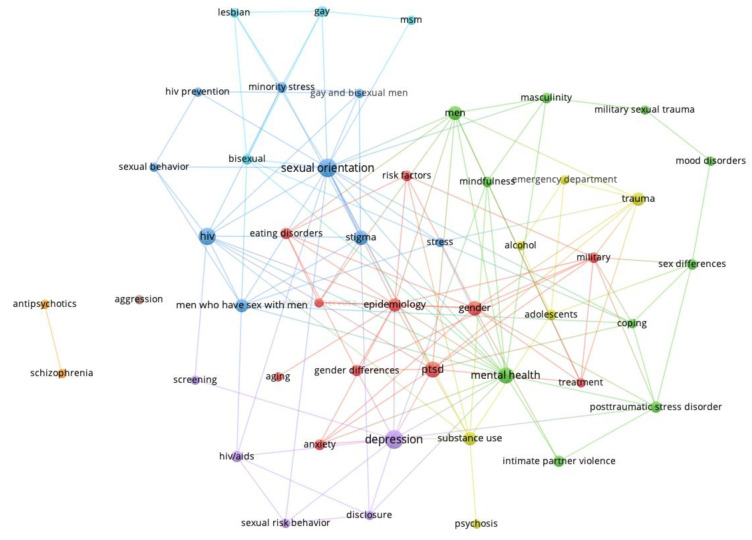

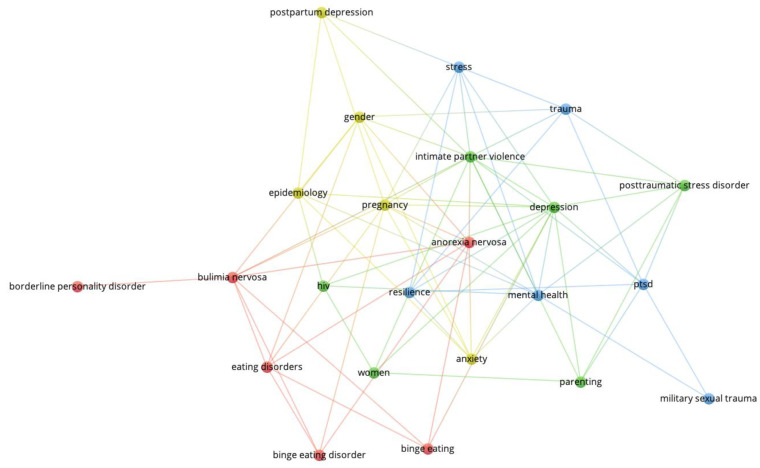
Keywords for men (**top** network) and women (**bottom** network) from 2015.

**Figure 2:**
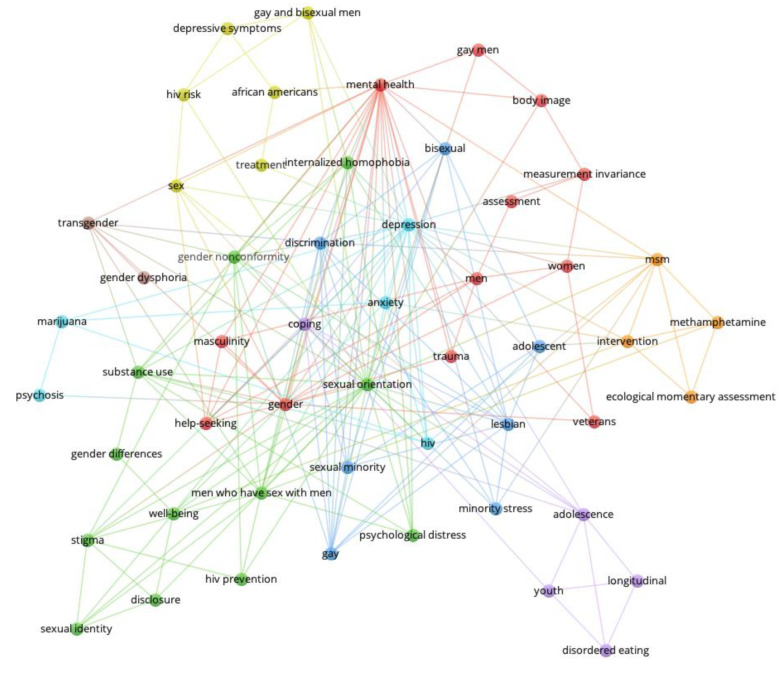

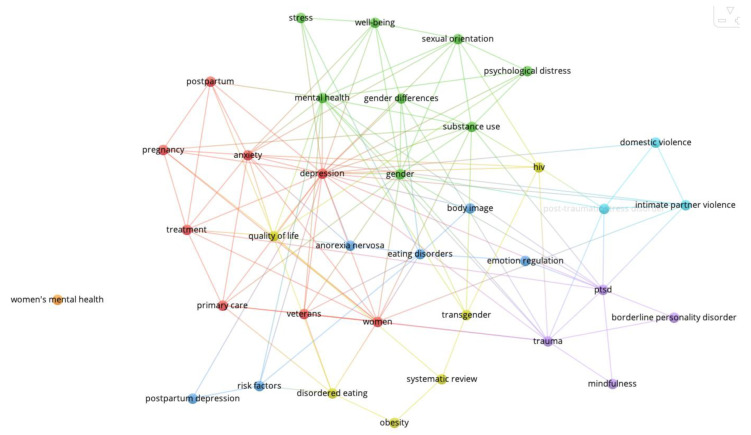
Keywords for men (**top** network) and women (**bottom** network) from 2018.

**Figure 3:**
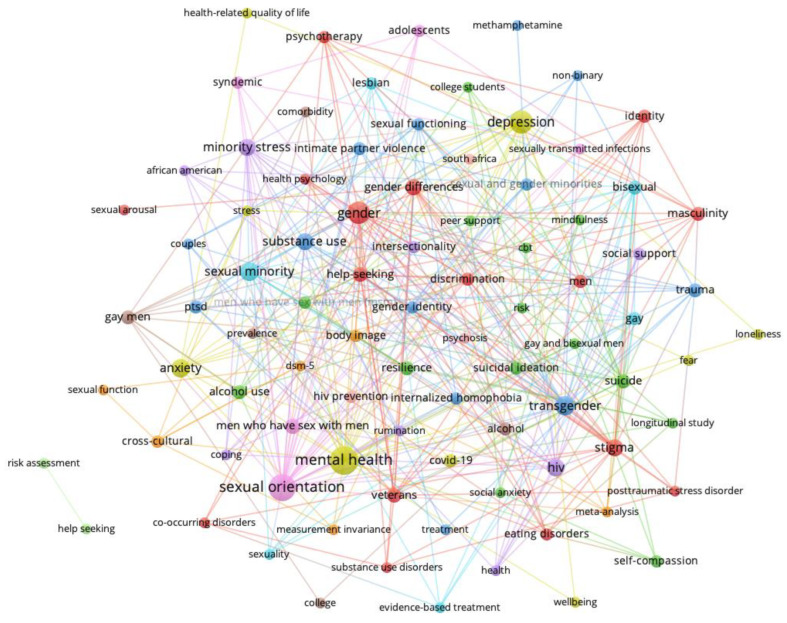

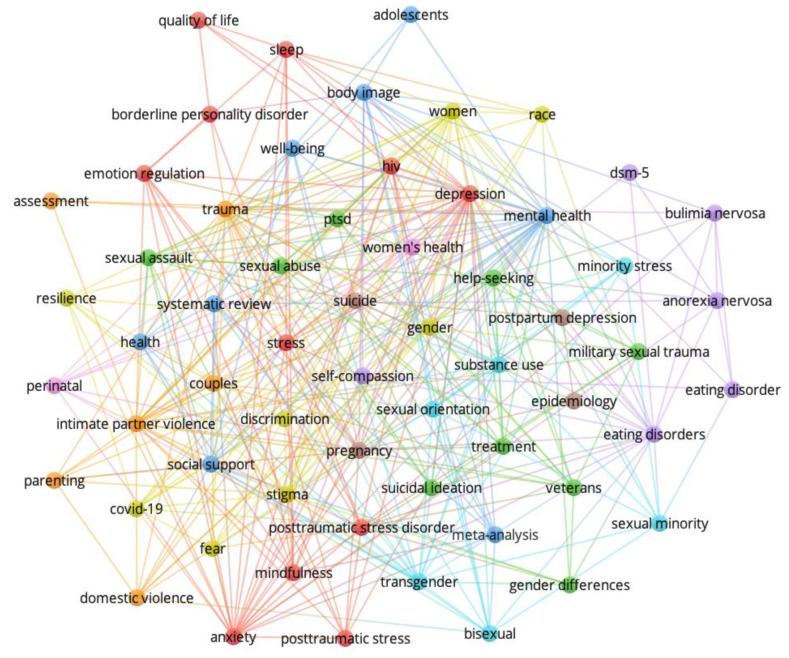
Keywords for men (**top** network) and women (**bottom** network) from 2020.

**Figure 4:**
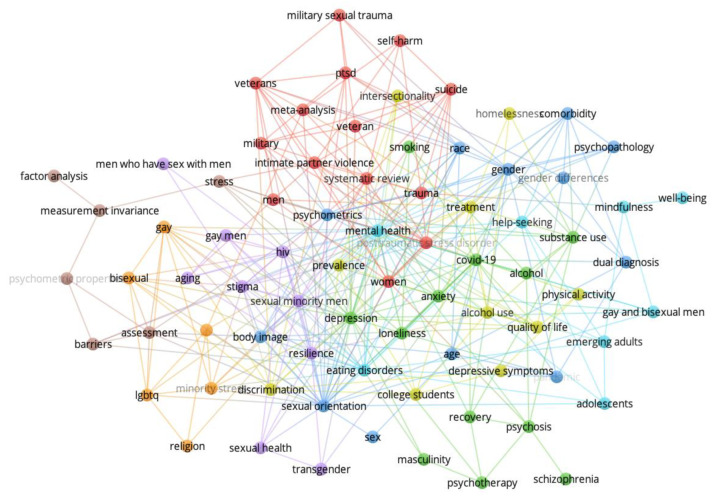

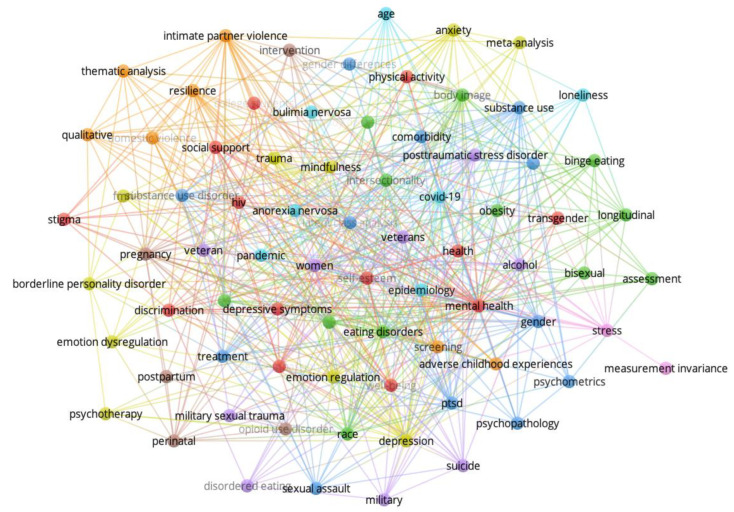
Keywords for men (**top** network) and women (**bottom** network) from 2022.

Figure 1, Figure 2, Figure 3 and Figure 4 show the network visualizations of the co-occurrence of keywords using the full counting method, meaning that the total number of occurrences of a term in all the documents is represented (with more frequently occurring terms represented by larger nodes). By contrast, the binary counting method assesses the number of documents in which a term occurs at least once (i.e., overall frequency of occurrence of a term is lost; [26]).

Figure 1 shows the keyword networks for men and women in 2015. Figure 2 shows the keywords networks for men and women in 2018. Figure 3 shows the keywords networks for men and women in 2020. Figure 4 shows the keywords networks for men and women in 2022. The clusters of different colored nodes and connections in Figure 1, Figure 2, Figure 3 and Figure 4 indicate that those words tended to co-occur with each other more often than they co-occurred with other terms in the network.

The layout parameters for each network were adjusted through trial and error in VOSViewer to display each network in such a way that the labels on each node were visible. Unfortunately, this approach contributed to the length of the line between two nodes being less informative (i.e., the thickness but not the distance of the connection conveys co-occurrence frequency) and in the clusters (i.e., nodes of the same color) being dispersed throughout the network. Other parameter settings will produce networks with nodes of the same color being more closely clustered together, but perhaps at the cost of label visibility. Larger nodes represent keywords that occurred frequently.

**Figure 5:**
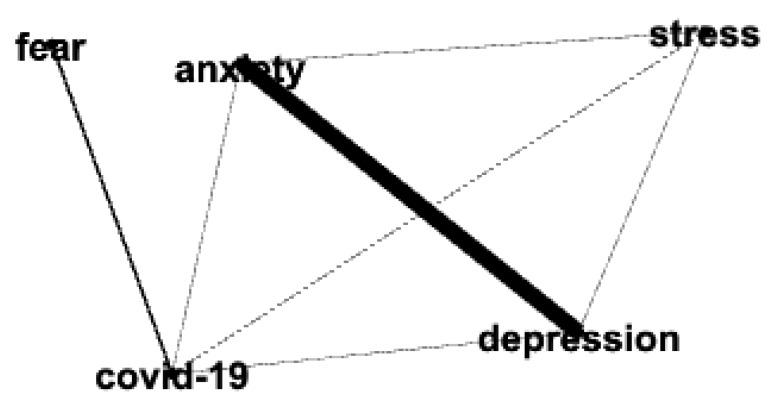

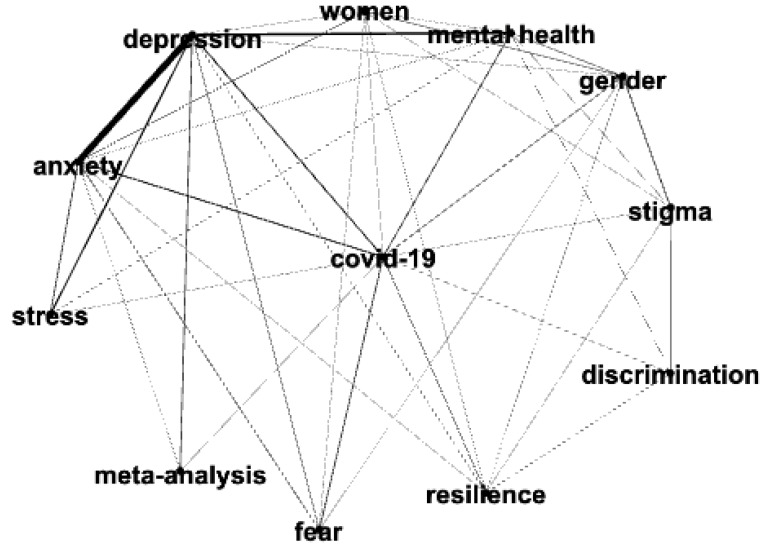
Ego network of the keyword COVID-19 for men (**top** network) and women (**bottom** network) in 2020.

**Figure 6:**
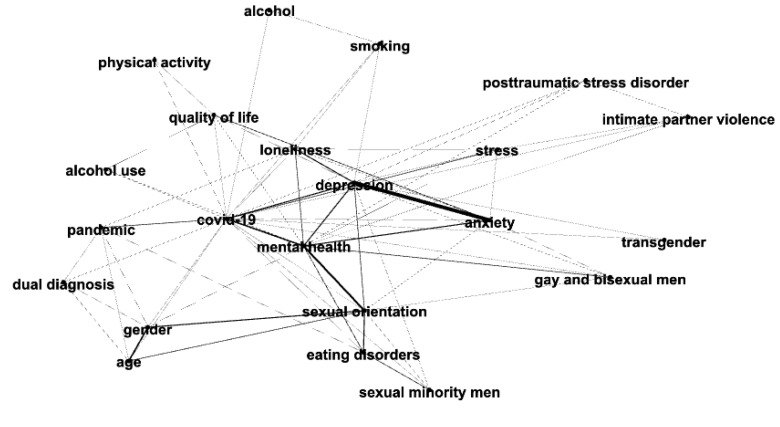

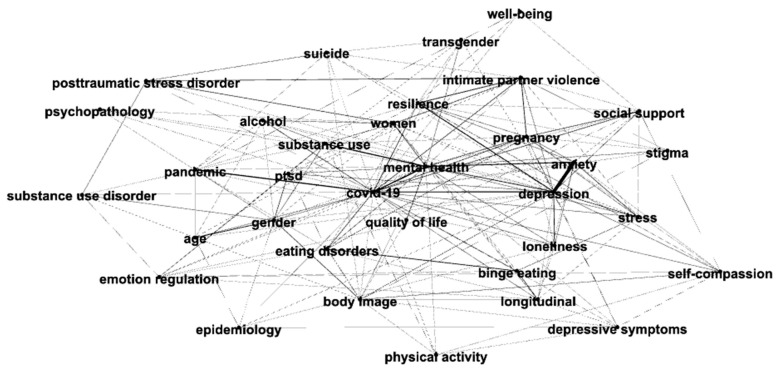
Ego network of the keyword COVID-19 for men (**top** network) and women (**bottom** network) in 2022.

Figure 5 shows the ego network of the keyword COVID-19 (and the keywords that co-occurred with it) for men and women in 2020. Figure 6 shows the ego network of the keyword COVID-19 (and the keywords that co-occurred with it) for men and women in 2022. The network analysis and visualization software Gephi (0.10.1) [30] was used to create the ego networks. 

## 4. Discussion

The present bibliometric analysis examined how research topics in Clinical Psychology journals were affected by the COVID pandemic. Other types of review articles typically summarize the current state of a particular research field or specific research topic, and perhaps compare effect sizes of various findings (e.g., [31,32]). Our use of conventional measures as well as network science methods in the present bibliometric analysis allowed us to examine not only how the broader research landscape changed in relation to the COVID pandemic (i.e., the macro-level, as in Figure 1, Figure 2, Figure 3 and Figure 4), but also how the COVID pandemic affected specific mental health research topics (i.e., the micro-level, as in Figure 5 and Figure 6). 

Prior to the start of the COVID-19 pandemic in 2020, various studies reported gender disparities in mental health issues and research (e.g., [24]). A similar gender disparity can be observed in Table 1 in the amount of mental health research published in Clinical Psychology journals on men and women (as well as in the research topics that are specific to men and women as shown in Table 2, Table 3, Table 4 and Table 5). In the years examined prior to the COVID pandemic (2015 and 2018), there were nearly twice as many research articles published that examined mental health in women than in men. Although the COVID pandemic exacerbated many pre-existing disparities in other areas of society (e.g., [4,5,6,7,8,9,10,11,12,13,14,15,16,33]), the values in Table 1 show that the number of published research articles that examined mental health in women and men remained at an approximately two-fold difference in the years examined after the start of the COVID pandemic (2020 and 2022). 

The disparity in the number of articles examining mental health in women and men remained relatively constant even though there was an overall increase in the amount of research published in Clinical Psychology journals in the years examined (from 2015 to 2022, there was a 1.69 increase in the number of articles). Note that the increase in the number of published articles in Clinical Psychology journals from 2015 to 2022 is within the range of what one would expect given the increase in scientific research in general [17]. 

We acknowledge that the timeframe (i.e., 2 years from the start to the end of the COVID pandemic) examined in the present study may be too short for a significant increase to be observed in the number of research articles published that examined mental health in women and men. Given that the time for scientific research to go through the peer-review process and ultimately be published has been estimated at 9 months (for STEM fields) to 18 months (for social sciences, arts/humanities, and business/economics; [34]) future work should re-examine the difference in the number of published research articles that examine mental health in women and men to determine if that disparity changes when viewed at a larger time frame (e.g., 5 or 10 years from the start of the COVID pandemic), and as the pandemic shifts to an endemic [35]. 

Turn now from the conventional measures of research output in Clinical Psychology journals, to the network analysis of author-provided keywords in articles published in Clinical Psychology journals in 2015, 2018, 2020, and 2022. As the label implies, author-provided keywords are terms or phrases that authors provide to summarize or categorize the associated article. Another type of “keyword” commonly used in bibliometric analysis is Keyword Plus terms, which are commonly occurring words or phrases extracted via an automatic computer algorithm from the titles of the references cited within an article [36]. Whereas Keywords Plus terms tend to be broader concepts (including research methods and techniques), author-provided keywords are often described as being (overly) specific concepts or terms [37]. Given the typical lag in publication (noted earlier) and the short time frame examined in the present case, we elected to use author-provided keywords to detect any rapidly occurring changes to the research landscape (i.e., too rapid to be detected in the terms that appear in the previously published works found in the reference section of recently published articles). 

To assess the overall research landscape in Clinical Psychology (i.e., the macro-level) and how it changed in response to the COVID pandemic, we created networks with author-provided keywords being represented as nodes and connections being placed between keywords that co-occurred in an article. As shown in Figure 1, Figure 2, Figure 3 and Figure 4 (also see Table 2, Table 3, Table 4 and Table 5), there are several terms that regularly occur only for men (e.g., *gay*, *men who have sex with men/msm*) and only for women (e.g., *postpartum*, *pregnancy*). There are also several terms that are common to mental health research on men and women, and that regularly occurred across the years examined: *anxiety*, *depression*, *gender*, *gender differences*, *hiv*, *stress*, *trauma*. The distinct presence of certain terms for only men and only women, as well as the perennial presence of fundamental terms in Clinical Psychology research for both men and women reassure us that the network science approach is indeed accurately depicting the research landscape.

Our confidence in this approach is increased by the term *COVID-19* appearing (as one would expect) in research on men and women only in 2020 and 2022. Also of interest, the term *fear* co-occurs with *COVID-19* (as shown in Figure 5) in research on men and women in 2020, but not in 2015 or 2018 (note that the term *fear* occurs in 2022, but fewer than the *x* = 3 occurrences for men and fewer than the *x* = 6 occurrences for women; therefore, it does not appear in our visualizations). Similarly, the term *pandemic* co-occurs with *COVID-19* (as shown in Figure 6) in research in men and women in 2022, but not in 2015 or 2018 (similar to the case for the term *fear*, the term *pandemic* occurred in 2020, but fewer than the *x* = 3 occurrences for men and fewer than the *x* = 6 occurrences for women; therefore, it does not appear in our visualizations). 

We now examine at the micro-level the term COVID-19. Figure 5 and Figure 6 show the ego networks for the term *COVID-19* and the terms that co-occur with it in 2020 and 2022, respectively. Note that the terms *anxiety*, *depression*, and *stress* appear and co-occur with the term *COVID-19* in the ego networks for Clinical Psychology research for both men and women in 2020 and 2022. In 2020, COVID-related research in men and women focused almost exclusively on the mental health topics of *anxiety*, *depression*, and *stress* (along with *fear*). This finding focused on research topics in Clinical Psychology journals complements the finding reported in [23] of the COVID pandemic resulting in a 25% increase in the global prevalence of anxiety and depression. 

After people had experienced the virus for some time and felt the influence of it in other parts of their lives [4,5,6,7,8,9,10,11,12,13,14,15,16], the ego network for *COVID-19* in 2022 shows that COVID-related research expanded beyond *anxiety*, *depression*, and *stress* to include some of the more fundamental topics of Clinical Psychology for both men and women, such as *eating disorders* and *post-traumatic stress disorder*, and to fundamental topics found only for research in men (e.g., *gay and bisexual men*) and to fundamental topics found only for research in women (e.g., *pregnancy*). Further, the ego network for *COVID-19* in 2022 (Figure 6) shows a number of research topics that may have been exacerbated by the quarantines and lockdowns experienced in many countries around the world during the pandemic (e.g., *alcohol use*, *substance use*, *intimate partner violence*, *loneliness*, and *physical activity*).

In addition to using ego networks to focus on the *COVID-19* keyword and co-occurring keywords, we also examined at the micro-level the most “important” keywords in the networks across the years that were sampled using common network science measures of centrality (i.e., degree centrality and closeness centrality [27]). In the present analysis (see Table 6), degree centrality counted how many keywords co-occur with a given keyword, whereas closeness centrality measured how many connections it takes (on average) to go via the shortest path from a given node to all other nodes in the network. Although *COVID-19* was not among the five nodes highest in degree centrality or closeness centrality in 2020, by 2022 the *COVID-19* keyword was among the 5 nodes highest in degree centrality and closeness centrality, suggesting that this research topic very quickly became an “important” topic in Clinical Psychology research and became interconnected to regularly occurring and fundamental topics of research in Clinical Psychology (e.g., *depression*).

Our findings at the macro- and micro-levels of the keyword networks regarding how COVID influenced research topics in Clinical Psychology journals also resemble the changes that have been observed in clinical practice during the COVID pandemic. For example, increases in the number of new patients were observed for many of the same conditions we observed in our networks of research keywords (e.g., anxiety, mood, and stress disorders), for clinicians engaging in in-person treatment [38] and in telehealth treatment [39]. The symmetry in the findings for clinical research and clinical practice lends additional credence to the novel methodology employed in the present analysis. 

Interestingly, although the COVID pandemic may have caused a shift in which research topics in Clinical Psychology received increased attention, the pre-existing differences in research on men and women found in certain research topics did not appear to change. Consider the topic of *eating disorders*. In 2015, this term co-occurred with *gender*, *gender differences*, *risk factors*, and *anorexia nervosa* for men, and *anorexia nervosa*, *binge eating (disorder)*, *gender*, and *bulimia nervosa* for women. In 2022, research on *eating disorders* in men expanded to include a wider range of populations (e.g., *adolescents*, *gay men*, and *race*), but still did not differentiate among different types of eating disorders to the same extent as is found in research on this topic in women. Research on *eating disorders* in women in 2022 also expanded to include a wider range of populations (e.g., *black women*, *bisexual*, and *race*), and continued to examine distinct types of eating disorders (e.g., *binge eating*, *bulimia nervosa*, *anorexia nervosa*, *disordered eating*, and *obesity*). In other words, the differences in research on *eating disorders* in men and women that existed prior to COVID remained after COVID. 

The present bibliometric analysis using methods from network science revealed at the macro-level and micro-level that the effects of COVID on Clinical Psychology research topics were uneven across research topics. Compare for example the differences in the keyword co-occurrence networks for research on *eating disorders* in men and women that existed pre-COVID and remained post-COVID, to the sharpened focus on the topics of *fear*, *anxiety*, and *depression* in 2020 (the year the COVID pandemic started). The methods from network science are being used increasingly not only for bibliometric analysis in the science of science, but are also being used increasingly in the psychological sciences to examine various aspects of cognition [40,41] and the symptoms associated with various psychopathologies such as eating disorders [42,43].

Future research employing another network analysis technique known as *textual forma mentis networks* [44] might prove useful for revealing finer details in the rapid changes that COVID might have brought about. For example, using textual forma mentis networks the researchers in [45] examined tweets from Italy and the US to extract how popular sentiment changed with the introduction of COVID-vaccines (as well as the frustrations when one vaccine was recalled). Because textual forma mentis networks can be created from larger selections of text (not simply co-occurring author-provided keywords as in the present study), future work could build textual forma mentis networks from the words found in the abstracts of published research articles to perhaps obtain finer-grained information about research topics, and perhaps detect changes that were too small to be detected with the approach employed in the present study. Such an approach may also circumvent the limitations imposed by using author-provided keywords (which tend to be overly broad, and in some cases must be selected from a restricted list of topics provided by publishers).

In addition to the limitations already described above, the present study focused just on journals that appeared in the Web of Science database with the “Clinical Psychology” designation. However, other professions also focus on mental health and have scientific journals in which research in those disciplines are published (e.g., Social Welfare, Counseling Psychology, etc.). Including journals from a broader range of mental health professions would have increased the generalizability and representativeness of our study. However, we do not believe that the general findings would have changed with the inclusion of journals from additional mental health fields. 

Similarly, our use of the search terms “men and mental health” and “women and mental health” in the WoS could have included alternative search terms (e.g., male, female, man, woman, etc.) in order to obtain a larger sample of publications. Inclusion of such alternative terms would likely have resulted in duplicate entries that would have been excluded from analysis, leaving us with a sample that probably would not have been much larger than the sample that was analyzed in the present study. 

The present analysis also used search terms related to biological sex (i.e., men and women). Future studies could employ the same methodology with search terms that focus on the social construct/identity of gender, and include nonbinary gender terms (e.g., genderqueer, gender-nonconforming, gender-neutral, agender, and gender-fluid). Despite not explicitly including search terms related to gender in the present analysis, several keywords related to sex, sexual orientation, and gender identity were nevertheless observed (see Table 2, Table 3, Table 4 and Table 5). 

## 5. Conclusions

Despite the limitations of the approach used in the present study, we have demonstrated that techniques from network science can be used to perform a bibliometric analysis that provides science administrators and policy makers, as well as individual researchers, with information about how the COVID pandemic affected research in Clinical Psychology. Whereas previous research examined how the pandemic affected the productivity of researchers [15,16], the present analysis examined how the pandemic affected the mental health topics in Clinical Psychology that researchers examined before, during, and after the pandemic. The methods and approach used in the present study, as well as other techniques from network science, may prove useful to uncover finer-grained details on this or related questions. 

## Figures and Tables

**Table 1:** Number (and percent) of articles obtained for the years examined.

Years	Men	Women	Total
2015	196 (35.4%)	357 (64.6%)	553
2018	225 (34.8%)	422 (65.2%)	647
2020	294 (32.6%)	607 (67.4%)	901
2022	295 (31.5%)	642 (68.5%)	937
Total	1010	2028	3038

Note: Men = “men and mental health”; women = “women and mental health”.

**Table 2:** Keywords that were common or unique to the networks for 2015.

Common Keywords	Men Keywords	Women Keywords
anorexia nervosa; anxiety; depression; eating disorders; epidemiology; gender; hiv; intimate partner violence; mental health; military sexual trauma; posttraumatic stress disorder; ptsd; stress; trauma	adolescents; aggression; aging; alcohol; antipsychotics; bisexual; coping; disclosure; emergency department; gay; gay and bisexual men; gender differences; hiv prevention; hiv/aids; lesbian; masculinity; men; men who have sex with men; msm; military; mindfulness; minority stress; mood disorders; psychosis; risk factors; schizophrenia; screening; sex differences; sexual behavior; sexual orientation; sexual risk behavior; stigma; substance use; treatment	binge eating; binge eating disorder; borderline personality disorder; bulimia nervosa; parenting; postpartum depression; pregnancy; resilience; women

**Table 3:** Keywords that were common or unique to the networks for 2018.

Common Keywords	Men Keywords	Women Keywords
anxiety; body image; depression; disordered eating; gender; gender differences; hiv; mental health; psychological distress; sexual orientation; substance use; trauma; treatment; veterans; wellbeing; women	adolescence; adolescent; african americans; assessment; bisexual; coping; depressive symptoms; disclosure; discrimination; ecological momentary assessment; gay; gay and bisexual men; gay men; gender dysphoria; gender nonconformity; help seeking; hiv prevention; hiv risk; internalized homophobia; intervention; lesbian; longitudinal; marijuana; masculinity; measurement invariance; men; men who have sex with men; msm; methamphetamine; minority stress; psychosis; sex; sexual identity; sexual minority; stigma; transgender; youth	anorexia nervosa; borderline personality disorder; domestic violence; eating disorders; emotion regulation; intimate partner violence; mindfulness; obesity; posttraumatic stress disorder; ptsd; postpartum; postpartum depression; pregnancy; primary care; quality of life; risk factors; stress; systematic review; transgender

**Table 4:** Keywords that were common or unique to the networks for 2020.

Common Keywords	Men Keywords	Women Keywords
adolescents; anxiety; bisexual; body image; couples; COVID-19; depression; discrimination; dsm; eating disorders; fear; gender; gender differences; health; help seeking; hiv; intimate partner violence; mental health; meta-analysis; mindfulness; minority stress; posttraumatic stress disorder; ptsd; resilience; self-compassion; sexual minority; sexual orientation; social support; stigma; stress; substance use; suicidal ideation; suicide; transgender; trauma; treatment; veterans; well-being	african american; alcohol; alcohol use; cbt; co-occurring disorders; college; college students; comorbidity; coping; cross-cultural; evidence-based treatment; gay; gay and bisexual men; gay men; gender identity; health psychology; health-related quality of life; help seeking; hiv prevention; identity; internalized homophobia; intersectionality; lesbian; loneliness; longitudinal study; masculinity; measurement invariance; men; men who have sex with men/msm; methamphetamine; non-binary; peer support; prevalence; psychosis; psychotherapy; risk; risk assessment; rumination; sexual and gender minorities; sexual arousal; sexual function; sexual functioning; sexuality; sexually transmitted infections; social anxiety; south africa; substance use disorders; syndemic	anorexia nervosa; assessment; borderline personality disorder; bulimia nervosa; domestic violence; eating disorder; emotion regulation; epidemiology; military sexual trauma; parenting; perinatal; postpartum depression; posttraumatic stress; pregnancy; quality of life; race; sexual abuse; sexual assault; sleep; systematic review; trauma; treatment; veterans; well-being; women; women’s health

**Table 5:** Keywords that were common or unique to the networks for 2022.

Common Keywords	Men Keywords	Women Keywords
age; alcohol; anxiety; assessment; bisexual; body image; college students; comorbidity; COVID-19; depression; depressive symptoms; discrimination; eating disorders; gender; gender differences; hiv; intersectionality; intimate partner violence; loneliness; measurement invariance; mental health; meta-analysis; military; military sexual trauma; mindfulness; pandemic; physical activity; posttraumatic stress disorder; ptsd; psychometrics; psychopathology; psychotherapy; race; resilience; sexual orientation; stigma; stress; substance use; suicide; transgender; trauma; treatment; veteran/veterans; well-being; women	adolescents; aging; alcohol use; barriers; dual diagnosis; emerging adults; factor analysis; gay; gay and bisexual men; gay men; help-seeking; homelessness; internalized homonegativity; lgbtq; masculinity; men; men who have sex with men; minority stress; prevalence; psychometric properties; psychosis; quality of life; recovery; religion; schizophrenia; self-harm; sex; sexual health; sexual minority men; smoking; systematic review	adverse childhood experiences; anorexia nervosa; binge eating; black women; body dissatisfaction; borderline personality disorder; bulimia nervosa; disordered eating; domestic violence; emotion dysregulation; emotion regulation; epidemiology; fmri; health; intervention; latent class analysis; longitudinal; obesity; opioid use disorder; perinatal; postpartum; pregnancy; qualitative; quality of life; screening; self-compassion; self-esteem; sexual assault; social support; substance use disorder; thematic analysis

**Table 6:** Centrality rankings for the top five and selected other author-provided keywords.

Degree Centrality							
2015		2018		2020		2022	
Men	Women	Men	Women	Men	Women	Men	Women
sexual orientation; mental health; epidemiology; depression; hiv	depression; intimate partner violence; pregnancy; mental health; anorexia nervosa	mental health; sexual orientation; depression; gender; discrimination	depression; mental health; women; anxiety; gender	sexual orientation; mental health; depression; gender; stigma; COVID-19 (62)	depression; mental health; trauma; intimate partner violence; women; COVID-19 (22)	mental health; sexual orientation; depression; COVID-19; eating disorders	mental health; depression; COVID-19; eating disorders; intimate partner violence
**Closeness Centrality**							
**2015**		**2018**		**2020**		**2022**	
**Men**	**Women**	**Men**	**Women**	**Men**	**Women**	**Men**	**Women**
antipsychotics; schizophrenia; sexual orientation; epidemiology; mental health	intimate partner violence; depression; pregnancy; mental health; anorexia nervosa	sexual orientation; mental health; depression; gender; discrimination	depression; mental health; women; gender; substance use	help seeking; risk assessment; sexual orientation; mental health; depression; COVID-19 (72)	depression; mental health; trauma; intimate partner violence; anxiety; COVID-19 (24)	mental health; sexual orientation; depression; COVID-19; eating disorders	mental health; depression; COVID-19; intimate partner violence; eating disorders

Note: Keyword terms are listed in order from “most” to “least”. The value in parentheses indicates the rank of the keyword if it was not in the list of top five keywords.

## Data Availability

The WoS files and .net files used to create the keyword networks and ego networks are available at an OSF website (https://osf.io/95yhd/).
